# Perception of Community Pharmacists in Malaysia About Mental Healthcare and Barriers to Providing Pharmaceutical Care Services to Patients with Mental Disorders

**DOI:** 10.1007/s10597-019-00496-4

**Published:** 2019-10-30

**Authors:** Yin Xuan Wong, Tahir Mehmood Khan, Zhi Jean Wong, Ab Fatah Ab Rahman, Sabrina Anne Jacob

**Affiliations:** 1 School of Pharmacy, Monash University Malaysia, Jalan Lagoon Selatan, 47500 Bandar Sunway, Selangor Malaysia; 2 Faculty of Pharmacy, Universiti Sultan Zainal Abidin, Besut Campus, 22200 Besut, Terengganu Malaysia; 3 Strathclyde Institute of Pharmacy and Biomedical Sciences, University of Strathclyde, 161 Cathedral St, Glasgow, G4 0RE Scotland, UK

**Keywords:** Pharmaceutical care, Community pharmacist, Mental health, Psychotropics, Malaysia

## Abstract

The aim of this study was to assess community pharmacists’ (CPs) perceptions toward mental healthcare, and the barriers faced in providing pharmaceutical care (PC) services to these patients. A 40-item survey was posted to CPs. Ninety-six pharmacists participated. The majority (84.2%) agreed there is a role for CPs to play in mental health care, while approximately 60% agreed it is their responsibility to provide PC to these patients. The biggest barrier to providing this service is the lack of knowledge, cited by close to 50% of respondents. This corresponds with the revelation that close to 60% believe that they have a poor or fair understanding of mental disorders. About 30% of respondents said they do not stock psychotropic drugs at all, mainly due to medico-legal reasons, and low prescription requests. Our findings highlight the need for more training of CPs in managing patients with mental disorders.

## Introduction

Mental disorders are among the strongest predictors of suicide, with more than 80% of patients committing suicide due to being untreated at the time of their death (Nock et al. 2009). Life expectancy rates of those with mental disorders are also 15 to 20 years less than the general population, often due to an increased risk in premature death (NHS England 2016). Nonadherence to medication among patients with mental disorders is reported to be more than 50% (Horne et al. 2009; Lacro et al. 2002; Lieberman et al. 2005), and leads to significantly negative clinical and economic consequences in these patients (Higashi et al. 2013; Ho et al. 2015). With the move towards deinstitutionalizing mental health care and encouraging management in the community setting, community pharmacists (CPs) can play a bigger role by providing pharmaceutical care (PC) services. A systematic review found that PC services by CPs resulted in improved adherence, and reduced the number of inappropriate medications prescribed in patients with mental disorders (Bell et al. 2005).

In Malaysia, a national survey revealed that 12% of adults suffer from some form of mental illness (Ministry of Health 2011), an increase from 10% in 1996 (Ministry of Health 1996). In the face of high costs of medications and budget cuts, restricted access to psychotropic medications could harm patients and result in poor treatment outcomes (Koyanagi et al. 2005). With the increasing prevalence of mental health disorders, there will be an increase in demand for psychotropics as well as pharmacists who are able to provide PC services to this group of patients. Community pharmacies can thus play an important role in ensuring patients have an interrupted supply of regular medications as they are highly accessible with extended opening hours.

Patients, however, have reported feeling stigmatized by healthcare professionals, encountering rejection and discrimination when seeking treatment (Knox et al. 2014). Studies have also found that CPs have a stigmatizing attitude toward patients with mental disorders (Liekens et al. 2012a, b). This is a profound scenario in Malaysia, with patients with schizophrenia, bipolar disorder, and major depressive disorder (MDD) stigmatized and discriminated the most by healthcare professionals (Hanafiah and Van Bortel 2015). Stigma is closely related to religion and culture, and has a strong influence on the views of healthcare professionals when dealing with these disorders and patients (Office of the Surgeon General (US), Center for Mental Health Services (US), and National Institute of Mental Health (US) 2001). In light of the cultural diversity in this country, which is made up of Malays (60%), Chinese (> 20%), Indians (~ 10%), and other native ethnic groups, we conducted this study to ascertain the perceptions of CPs and the barriers faced in providing PC services to patients with mental disorders.

## Methods

This was a cross-sectional study involving CPs in Malaysia. A list of 926 pharmacies registered in Peninsular Malaysia was obtained from the Malaysian Pharmaceutical Society website. Assuming an overestimated response distribution of 50%, a minimum effective sample size of 88 was needed to achieve a confidence interval of 95% and a 10% margin of error (Raosoft 2004). Taking into account the low response rate of surveys, 300 pharmacies were randomly selected. A survey containing a cover letter inviting pharmacists to take part in the study and a pre-paid return envelope was mailed to those community pharmacies. No financial incentives were offered, and no reminders were sent.

### Study Instrument

The survey form was a 40-item questionnaire consisting of both open-ended and close-ended questions. Some of these responses were rated on a 5-point Likert scale ranging from 1 (strongly disagree) to 5 (strongly agree). The following areas were included: perception of pharmacists on patients and mental disorders, knowledge on mental health, the level of comfort regarding their level of confidence and responsibilities managing mental disorders, PC services provided (including types of psychotropics stocked and prescription for psychotropics), reasons for not stocking, and barriers to providing PC services. Demographics such as qualifications and work experience were also obtained from all respondents.

The survey was developed based on the study objectives as well as a review of the literature, and 10 experts in the fields of mental health as well as PC performed face and content validation. The survey was pilot-tested on five pharmacists to assess their comprehension of the survey, and the time taken to complete it. The survey took approximately 20–30 min to complete, and was available in English. This study was approved by the University Ethics Committee. There are no known conflicts of interest, and all authors certify responsibility for the manuscript.

### Data Analysis

Baseline demographic data were presented using descriptive statistics. Continuous variables were expressed by means and standard deviations, whereas categorical/nominal data were presented using frequency and percentage. Content analysis was performed on the open-ended comments. To create a composite picture on what respondents disagreed and agreed on for questions employing the 5-point Likert scale responses, the scores for the first two columns (“strongly disagree” and “disagree”) were added up to show what they disagreed on, while the scores for the last two columns (“agree” and “strongly agree”) were totaled up to show what they agreed on. Likert-scale responses for (1) the perception of pharmacists with regard to mental illness and patients with mental disorders, and (2) the level of comfort of pharmacists in managing mental disorders were also tabulated as means, and totaled to give an overall picture. The level of comfort of pharmacists encompassed the responses of pharmacists in reference to their level of confidence and responsibility with regard to managing mental disorders.

General linear regression was employed to examine predictors of perceptions and level of comfort of pharmacists, and the following independent variables were included in the models: age, gender, type of pharmacy, knowledge level, personal and family history of mental disorders, years since graduation, postgraduate qualifications, work experience in community and hospital pharmacies, and belief if personal opinion about mental illness will affect their ability to provide PC to patients. Dummy variables were created for categorical variables with more than two outcomes. Variance inflation factor values were inspected for the possibility of multicollinearity, with results higher than 10 being considered as indicative of this problem. The a priori level of significance was 0.05, and all analyses were performed using Microsoft Excel and SPSS 24.0 statistical software (SPSS, Inc., Chicago, IL, USA).

To determine the potential barriers in providing PC, the relative importance weight factor (RIWF) was calculated for each of the 16 barriers identified to determine the top three barriers, with the value closest to ‘1’ signifying the main barrier. Calculation of the RIWF was done according to the following equation:

$${\text{RIWF}} = \frac{{\varSigma {\text{W}}\;\left( {0 \le {\text{RIWF}} \le 1} \right)}}{{{\text{A}} \times {\text{N}}}},$$

where W is the weight given to each factor by the respondents and ranges from 1 to 5 (where ‘1’ is ‘strongly disagree’ and ‘5’ is ‘strongly agree’), A is the highest weight i.e. 5, and N is the total number of respondents (Gündüz et al. 2013).

## Results

### Demographics of Respondents

Of the 300 pharmacies, 96 pharmacists responded to the survey, while 39 envelopes were returned because the premises were no longer in business. There was a slight female preponderance (52.1%). Most of the respondents had undergraduate-level education (70.8%), and less than 5 years of working experience in a community pharmacy (42.7%) or hospital (67.7%). None of the respondents believed they possessed excellent knowledge of mental disorders (Table 1). The majority agreed that the main reasons for selecting ‘Poor’ or ‘Fair’ knowledge were ‘inadequate training in this field’ (93%), and ‘few prescriptions for psychotropics’ (87.3%). Others included ‘inadequate material on mental health therapy’ (54.5%), ‘inadequate education during undergraduate on mental disorders’ (52.6%), ‘perceive mental disorders as a difficult area or specialty compared to cardiovascular and endocrine disorders such as diabetes’ (51.8%), and ‘lack of interest’ (26.8%).

**Table 1:** Characteristics of respondents (n = 96)

Characteristics	N (%)
Age, years (mean ± SD)	35.33 ± 12.94
Ethnicity	
Malay	19 (19.8)
Chinese	67 (69.8)
Indian	9 (9.4)
Other	1 (1.0)
Years after graduation (basic degree) (n = 95)	
< 5 years	29 (30.5)
5–10 years	19 (20.0)
> 10 years	47 (49.5)
Role in pharmacy	
Owner	23 (24.0)
Manager	12 (12.5)
Full time pharmacist	39 (40.6)
Locum pharmacist	27 (28.5)
Type of pharmacy (n = 94)	
Chain	33 (35.1)
Independent	61 (64.9)
Personal history of mental illness	
Yes	3 (3.1)
No	88 (91.7)
Preferred not to answer	5 (5.2)
History of mental illness in a family member/close friend	
Yes	25 (26.0)
No	64 (66.7)
Preferred not to answer	7 (7.3)
Knowledge level on mental disorders	
Good	34 (37.4)
Fair	49 (53.8)
Poor	8 (98.8)
Belief that own opinions and beliefs about mental disorders would affect their ability to provide PC to these patients	
Definitely	20 (20.8)
Probably	27 (28.1)
Maybe	23 (24.0)
Not at all	18 (18.8)
Don’t know	8 (8.3)

*SD* standard deviation

### Stocking and Pharmaceutical Care Services

Thirty-one (32.3%) pharmacists did not stock any psychotropics at all, and 40% of these referred patients with mental disorders to hospitals, followed by private clinics (27.1%), and other community pharmacies (15.6%). Reasons for not stocking were strict regulations of record keeping as required by law (72.4%), numerous medical legal issues (69%), and that they only received few prescription requests (60.7%) (Table 2). The maximum number of psychotropics stocked was 29, as reported by 2 (2.1%) pharmacists, and antidepressants were the class of psychotropics most stocked (48.7%). Of those who stocked psychotropics, the majority stocked carbamazepine (70.8%), followed by amitriptyline and escitalopram (64.6% respectively), and valproic acid (56.9%) (Table 3). Prescriptions for psychotropics were most frequently for sleep-related disorders (37.5%), followed by depression (21.9%) and anxiety (18.8%). Prescribers were generally evenly spread between general practitioners (27.1%), and specialists from both government (28.1%) and private (29.2%) hospitals/institutions.

**Table 2:** Reasons for not stocking psychotropics (n = 29)

Statement	Disagree, N (%)	Neither agree nor disagree, N (%)	Agree, N (%)
(a) Because mentally ill patients have unpredictable behaviour and are aggressive; and as such I’d rather not deal with them	11 (37.9)	8 (27.6)	10 (34.5)
(b) I fear for my life and as a result I’d rather not deal with these patients	16 (55.2)	7 (24.1)	6 (20.7)
(c) Fear of social drug-related complications	9 (31.0)	10 (34.5)	10 (34.5)
(d) I feel uncomfortable stocking psychiatric medications for fear of being robbed (n = 28)	15 (53.6)	7 (25.0)	6 (21.4)
(e) Numerous medical legal issues	3 (10.3)	6 (20.7)	20 (69.0)
(f) Tough regulations of record keeping as required by law	4 (13.8)	4 (13.8)	21 (72.4)
(g) Unfamiliar with psychotropics	12 (41.4)	11 (37.9)	6 (20.7)
(h) Unfamiliar with mental disorders	5 (17.2)	13 (44.8)	11 (37.9)
(i) Few requests (n = 28)	3 (10.7)	8 (28.6)	17 (60.7)
(j) Determined by parent company/boss/manager (n = 28)	7 (25.0)	11 (39.3)	10 (35.7)

**Table 3:** Stocking and pharmaceutical care services (n = 96)

Statement	N (%)
Frequency of prescription requests for psychotropics	
Daily	2 (2.1)
Two to three times	7 (7.3)
Once a week	22 (22.9)
Once a month	22 (22.9)
Once in 3–6 months	28 (29.2)
Once a year	7 (7.3)
Never	8 (8.3)
Gender of the majority of patients with prescription requests for psychotropics	
Male	31 (32.3)
Female	15 (15.6)
Equal	30 (31.3)
Age group of the majority of patients with prescription requests for psychotropics	
18–29	3 (3.1)
30–39	12 (12.5)
40–49	30 (31.3)
50–59	25 (26.0)
60–69	16 (16.7)
70–79	1 (1.0)
Average number of psychotropics sold a month (n = 61)	
0	26 (42.6)
1	7 (11.5)
2	5 (8.2)
3	11 (18.0)
4	1 (1.6)
5	6 (9.8)
> 5	5 (8.2)
Pharmaceutical care services provided to patients with mental disorders^a^	
Counseling	58 (60.4)
Dispensing of drugs	45 (46.9)
Provision of drug information	37 (38.5)
Monitoring for side effects	16 (16.7)
Monitoring for adherence	10 (10.4)
Solving drug-related problems	12 (12.5)
None	24 (25.0)
Monitoring of efficacy	7 (7.3)
Frequency of providing drug counseling to patients, or relatives of patients with mental disorders	
Daily	1 (1.0)
Two to three times	4 (4.2)
Once a week	8 (8.3)
Once a month	22 (22.9)
Once in 3–6 months	34 (35.4)
Never	27 (28.1)
Psychotropics being stocked (n = 719)	
Antidepressants	
SSRIs	139 (19.3)
TCAs	69 (9.6)
SNRIs	30 (4.2)
Others	98 (13.6)
Antipsychotics	
Atypicals	107 (14.9)
Typicals	48 (6.8)
Anxiolytic	
Benzodiazepines	30 (4.2)
Others	40 (5.6)
Drugs for bipolar disorder/antimanic^b^	146 (20.3)

*SSRI* selective serotonin reuptake inhibitor, *TCA* tricyclic antidepressant, *SNRI* serotonin–norepinephrine reuptake inhibitors, *MAOI* monoamine oxidase inhibitors

^a^Respondents were allowed to select more than one response, therefore the sum may not always be 100%

^b^Includes antiepileptics and lithium

The majority (84.2%) of respondents agreed that there is a role for CPs to play in mental health care, while 61.7% agreed it is their responsibility to provide PC to patients with mental disorders. However, 66% agreed that only pharmacists with knowledge and training in mental health care should handle such patients. Counseling was the main PC service provided, as reported by 60.4% of respondents. The majority of respondents, however, reported low frequencies of providing PC services, with less than 20% monitoring for efficacy, side effects, and adherence (Table 3).

### Perception and Level of Comfort of Pharmacists in Managing Mental Disorders

While 80% agreed that mental illness is nothing to be ashamed of, respondents were overall neutral with regard to their perception of patients with mental disorders, and of mental illness (Table 4). A general linear regression was calculated to predict the perception of pharmacists based on the independent variables. The model reasonably fit well, model assumptions were met, and there were no multicollinearity problems. A significant regression equation was found [F(5, 89) = 3.054, p = 0.01], with an R^2^ of 0.146. The perception of pharmacists is equal to 2.799 + 0.298 (Ethnicity1), where [Ethnicity1] is coded as 0 = Malay, 1 = Chinese, 0 = Indian, 0 = Others. Chinese pharmacists had more positive perceptions compared to Malay pharmacists about patients with mental disorders, and about mental illness (*p* = 0.02). Respondents were neutral with regard to their confidence level and responsibility in managing mental disorders (Table 5). General linear regression found no significant relationship between level of comfort and the independent variables.

**Table 4:** Perception of pharmacists on patients with mental disorders and mental illness

Statement	Mean (± SD)	Disagree, N (%)	Neither agree nor disagree, N (%)	Agree, N (%)
(a) I find patients with mental disorders easily approachable (n = 95)	2.78 (0.84)	33 (34.7)	45 (47.4)	17 (17.9)
(b) Handling of patients with mental disorders is easier compared to patients with other diseases (n = 94)	2.46 (0.77)	51 (54.3)	35 (37.2)	8 (8.5)
(c) Mental illness is nothing to be ashamed of (n = 94)	4.04 (0.83)	6 (6.4)	12 (12.8)	76 (80.9)
(d) All patients with mental disorders are potentially dangerous to the people around them (n = 95)	3.26 (0.93)	42 (44.2)	29 (30.5)	24 (25.3)
(e) I am not afraid of patients with mental disorders (n = 93)	3.22 (0.81)	18 (19.4)	38 (40.9)	37 (39.8)
(f) Patients with mental disorders do not want to talk to a pharmacist about their mental health symptoms (n = 95)	2.99 (0.88)	28 (29.5)	37 (38.9)	30 (31.6)

**Table 5:** Level of comfort of pharmacists in managing mental disorders

Statement	Mean (± SD)	Disagree, N (%)	Neither agree nor disagree, N (%)	Agree, N (%)
(a) I find it easy to deal with relapse and non-adherence in patients with mental disorders (n = 95)	2.57 (0.79)	41 (43.2)	45 (47.4)	9 (9.5)
(b) I have an interest in providing pharmaceutical care to patients with mental disorders (n = 94)	3.30 (0.84)	17 (18.1)	35 (37.2)	42 (44.7)
(c) I have enough knowledge on the pharmacotherapy of patients with mental disorders (n = 94)	2.78 (0.81)	34 (36.2)	44 (46.8)	16 (17.0)
(d) I feel confident enough to provide pharmaceutical care to patients with mental health problems (n = 93)	3.19 (0.89)	19 (20.4)	38 (40.9)	36 (38.7)
(e) I feel comfortable enough to provide pharmaceutical care to patients with mental health problems (n = 94)	3.35 (0.92)	18 (19.1)	27 (28.7)	49 (52.1)
(f) There is enough motivation for me to provide pharmaceutical care to patients with mental disorders (n = 95)	3.16 (0.90)	22 (23.2)	40 (42.1)	33 (34.7)
(g) I feel comfortable asking patients their reason(s) for using psychotropics (n = 95)	3.43 (0.81)	15 (15.8)	28 (29.5)	52 (54.7)
(h) I feel comfortable discussing the symptoms of mental illness with patients (n = 95)	3.37 (0.89)	16 (16.8)	29 (30.5)	50 (52.6)
(i) I received adequate education/training about mental health during my undergraduate pharmacy education (n = 95)	2.83 (1.01)	38 (40.0)	30 (31.6)	27 (28.4)

### Perceived Barriers in Providing Pharmaceutical Care to Patients with Mental Disorders

According to rankings based on RIWF, the most frequently cited barrier was the lack of knowledge about mental disorders. Others included patients’ lack of understanding about PC and legal requirements (Table 6). There were 43 open-ended comments with regard to what would facilitate the provision of PC to patients with mental disorders, where 23.3% of respondents mentioned the need for training not just with regard to mental disorders, but also counseling and PC. Four commented about the importance of having details on the patient history, while two mentioned the need for dispensing separation, collaboration between doctors and pharmacists, and social awareness to break the stigma.

**Table 6:** Barriers to providing pharmaceutical care to patients with mental disorders in community pharmacy

Statement	Disagree, N (%)	Neither agree nor disagree, N (%)	Agree, N (%)	RIWF	Rank
(a) Lack of knowledge about mental disorders (n = 92)	23 (25.0)	24 (26.1)	45 (48.9)	0.692	1
(b) Patients do not understand pharmaceutical care (n = 93)	8 (8.6)	28 (30.1)	57 (61.3)	0.690	2
(c) Lack of training in pharmaceutical care practice (n = 92)	13 (14.1)	28 (30.4)	51 (53.1)	0.660	6
(d) Insufficient staff (n = 89)	20 (22.5)	23 (25.8)	46 (51.7)	0.613	8
(e) Lack of communication skills (n = 92)	39 (42.4)	31 (33.7)	22 (23.9)	0.542	14
(f) Lack of drug information sources (n = 90)	45 (50.0)	25 (27.8)	20 (22.2)	0.504	16
(g) Lack of documentation skills (n = 92)	30 (32.6)	34 (37.0)	28 (30.4)	0.571	13
(h) Lack of demand from patients (n = 92)	14 (15.2)	26 (28.3)	52 (56.5)	0.671	5
(i) Lack of private space/counseling area (n = 90)	27 (30.0)	22 (24.4)	41 (45.6)	0.598	11
(j) Lack of encouragement from head office/manager (n = 91)	27 (29.7)	39 (42.9)	25 (27.5)	0.541	15
(k) Lack of initiative (n = 92)	22 (23.9)	39 (42.4)	31 (33.7)	0.604	10
(l) Legal requirements (n = 92)	15 (16.3)	24 (26.1)	53 (57.6)	0.685	3
(m) Fear or discomfort of dealing with psychiatric patients (n = 91)	30 (33.0)	28 (30.8)	33 (36.3)	0.579	12
(n) Patient factors associated with their symptoms e.g. cognitive dysfunction, hostility, inattentiveness, irritability (n = 92)	20 (21.7)	38 (41.3)	34 (37.0)	0.606	9
(o) Time constraints (n = 92)	21 (22.8)	20 (21.7)	51 (55.4)	0.646	7
(p) Lack of patient information e.g. drug indication, treatment goals, etc. (n = 92)	14 (15.2)	22 (23.9)	56 (60.9)	0.675	4

## Discussion

Our survey has attempted to determine the involvement of CPs in providing PC to patients with mental disorders. Our findings suggest that while CPs agree they have a role in managing mental disorders in the community, there are barriers to providing this, the main one being their lack of knowledge. Indeed, only slightly more than 30% of respondents stated that they have a good understanding of mental disorders. This should be expected since many seldom handled prescriptions on psychotropic medications, and therefore were less likely to be exposed to such patients. Approximately 30% of pharmacies also do not stock psychotropics.

### Stocking and Pharmaceutical Care Services

Strict regulations of record keeping was one of the main reasons for not stocking psychotropics. A report by the International Narcotics Control Board involving 102 countries found that the stocking of such substances were discouraged due to the strict restrictions regarding the dispensing protocol, and the time it consumed (International Narcotics Control Board 2016). Similarly in Saudi Arabia a survey showed that the commonly prescribed psychotropic drugs were unavailable in more than half of the 248 community pharmacies studied due the strict regulations for purchasing and dispensing psychotropics imposed by the government (Al-Ruthia et al. 2017).

The low frequency of prescription requests is another contributing factor, where close to 30% of the respondents revealed that they had never dispensed psychotropics before. This is because the community pharmacy is often regarded as being placed in the primary care setting, with the pharmacist perceived as playing a peripheral as opposed to a central role, in the healthcare system (Crump et al. 2011). In Malaysia the majority of patients seek treatment at government hospitals, which are provided free of charge or at minimal cost, and only purchase psychotropics at community pharmacies when government hospitals are out of stock or do not stock the psychotropics prescribed, similar to that observed in Ghana (Owusu-Daaku et al. 2010). The absence of dispensing separation in Malaysia is also a possible factor for low requests (Shafie et al. 2012), as most medications are dispensed from government and private hospitals and clinics. As a result, most community pharmacies rarely receive prescriptions (Nik et al. 2016; Shafie et al. 2012).

It is encouraging to find out that the majority of respondents agreed that there is a role for CPs in mental health care. This mirrored findings in the United States (US) and Canada, where CPs demonstrated positive attitudes toward treating patients with mental disorders (Guillaumie et al. 2015; Watkins et al. 2017). In a roundtable discussion organized by the Royal Pharmaceutical Society in the United Kingdom (UK) to discuss how pharmacists can support patients with mental health issues, pharmacists expressed excitement in getting more involved in the care of this subset of patients. It was also highlighted that CPs have a significant role to play in the early identification of people with mental disorders, as well as in improving the physical health and adherence rates of these patients (Royal Pharmaceutical Society 2018).

While 60% of respondents reported providing counseling to patients, the majority reported low frequency of providing these services. This could be due to the low frequency of prescription requests. Additionally, less than 20% of respondents reported monitoring for side effects, efficacy, and adherence. As previously mentioned, nonadherence is a significant issue in this population (Higashi et al. 2013; Ho et al. 2015), with side effects cited as a main factor (De Las Cuevas et al. 2014; Hung et al. 2011). There is, however, a misconception on the part of health professionals that the newer generation drugs are void of side effects, thus scant attention is paid to this (Lingam and Scott 2002; Pampallona et al. 2002).

### Perception and Level of Comfort of Pharmacists in Managing Mental Disorders

Since Malaysia is a multiracial country, it provided us with the opportunity to look at how different races respond to this issue. Chinese pharmacists had significantly more positive perceptions compared to Malay pharmacists about patients with mental disorders, and about mental illness. It has indeed been found that Malays are more likely to reject mental illness as they believe the condition is caused by supernatural forces (Hanafiah and Van Bortel 2015), making them more predisposed to seeking spiritual and religious therapies (Khan et al. 2010). Subsequently, we found that the majority were quite neutral with regard to their confidence level and responsibility in managing patients with mental disorders. This is not an encouraging trend if PC services to patients with mental disorders are to be provided.

### Perceived Barriers in Providing Pharmaceutical Care to Patients with Mental Disorders

Lack of knowledge about mental disorders was ranked as the main barrier, which corresponds with the findings where close to 60% of the respondents rated their knowledge of mental disorders as ‘fair’ or ‘poor’. This corroborates with studies involving CPs in Australia, Canada, Ghana, Belgium, the UK, and the US, who have cited their low confidence and discomfort due to their lack of training, education, and clinical skills in mental disorders; as barriers to them monitoring patients, identifying drug-related problems, and discussing symptoms of mental disorders as well as drug-related issues with patients (Cates et al. 2005; Liekens et al. 2012a, b; Maslen et al. 1996; O’Reilly et al. 2015; Owusu-Daaku et al. 2010; Phokeo et al. 2004; Watkins et al. 2017).

A large number of respondents agreed that patients do not understand the concept of PC, mirroring findings from the Middle-East, Asia, and rural areas in the UK, which found that the majority of consumers have a negative view of CPs, perceiving them to be more business-centric, and declaring that their primary role is to dispense medication (Awad et al. 2017; Merks et al. 2016). Indeed very few are aware of the expanded roles of CPs such as in providing PC services and managing chronic diseases (Hasan et al. 2015; Merks et al. 2016; Erah and Chuks-Eboka 2008; You et al. 2011), and still defer to doctors as the primary expert on drug-related matters (Awad et al. 2017; Hallak 2012). It is postulated that in these countries, similar with Malaysia, consumers are more comfortable with the traditionally-defined roles of healthcare professionals in the healthcare system, where doctors are held in high-esteem on all health-related matters. This then leads them to reject the clinical roles of pharmacists. Additionally, as patients usually receive their prescribed medications from doctors, they are unaware of pharmacists’ roles beyond dispensing medication (You et al. 2011).

### Implications for Future Training, Policy and Practice

The World Health Organization (WHO) has highlighted that CPs should provide PC services to patients (World Health Organization 1994). CPs are the most accessible healthcare professionals due to their proximity to patients, and subsequently the high volume of customers who frequent their pharmacies (Awad et al. 2017; Rubio-Valera et al. 2014; World Health Organization 1994). Pharmacists have also expressed a desire to expand their roles beyond dispensing, and have made the move to engage in more clinically-oriented roles (Luetsch 2017; Murphy et al. 2016a), while patients have expressed that they find pharmacists more approachable and accessible than doctors (Deslandes et al. 2015). CPs are in the perfect position to screen for depression, provide counseling, monitor and encourage adherence, and monitor the outcomes of pharmacotherapy (Rubio-Valera et al. 2014). Findings from this study, however, indicate that there are a few barriers to CPs providing PC services to these patients, such as gaps in knowledge and training, the lack of public knowledge on PC services, and the influence of culture and stigma.

CPs have requested more education and training programs in mental health, such as training in psychosocial communication (Liekens et al. 2012a, b), or regular workshops to educate them on counseling, therapeutics, and signs and symptoms of mental disorders (Owusu-Daaku et al. 2010). It was indeed found that CPs who attended continuing education courses on mental disorders were more confident in interacting with these patients (Maslen et al. 1996), and the quality of pharmacist–patient interactions was improved (Liekens et al. 2014). Additionally, there is a need for mental health education modules to adopt methods which will reduce stigma, instead of solely focusing on the science and therapeutic aspects of the diseases (Murphy et al. 2016b; Rubio-Valera et al. 2014). Incorporating Mental Health First Aid (MHFA) training in pharmacists’ training is also proposed (Kirschbaum et al. 2016).

Approximately 60% of respondents agreed that members of the community are also ignorant about the clinical role of CPs, and one reason for this could be the lack of dispensing separation. This has also led to the lack of prescriptions received, leading to minimal to zero interactions between CPs and patients with mental disorders, curbing their opportunity to learn on the job and enhance their psychosocial communication skills. More importantly, this has denied CPs the chance to provide PC services. Dispensing separation is practiced in many Western countries and has proven to be effective in preventing over-prescribing, minimizing prescription errors or misuse of medication, as well as reducing health-related costs (Salmasi et al. 2016). A strong opposition to this separation was the lack of pharmacists, however it is estimated that Malaysia will soon reach the pharmacist-to-population ratio as recommended by the WHO (Shafie et al. 2012). Patients themselves acknowledge pharmacists as drug experts—viewing them as more suitable to dispense compared to doctors, and willing to pay for their services (Shafie and Hassali 2010). It is thus, time for Malaysia to adopt dispensing separation to ensure the optimum care of these patients in the community.

### Limitations and Strengths

Our study had a number of limitations such as recall bias especially on questions related to the number and type of patients. Given the low response rate of 36.8%, findings might not be representative of the perceptions of all CPs in Peninsular Malaysia. However, the number of responses was greater than the 88 required. In addition, based on previously conducted nationwide studies of CPs in Malaysia, the demographics of respondents in our survey seem to be a fairly representative sample of CPs in Malaysia, where the majority are Chinese, female, and between 31 and 40 years of age (Chong et al. 2010; Khan et al. 2016; Siang et al. 2008). Other than that, as the question on the stocking of psychotropics was the first question, the majority of CPs might have chosen not to respond to the rest of the questions as they did not stock any psychotropics, assuming the survey was not relevant to them.

## Conclusions

Many CPs are positive toward their involvement in mental healthcare. The main barrier to providing PC, however, is the lack of knowledge. About a quarter of the respondents claimed that they did not stock any psychotropics due to legal issues and minimal prescription requests. Findings can be used by the relevant healthcare authorities in the design and implementation of programs and workshops targeted toward training CPs. This will hopefully encourage more community pharmacies to sell psychotropics and provide PC to this subset of patients, which will subsequently result in patients who are able to function in society and contribute to the economy.
